# 1-Methyl-3-phenyl­thio­urea

**DOI:** 10.1107/S1600536814007442

**Published:** 2014-04-09

**Authors:** Hou-xiang Su

**Affiliations:** a Lvliang University, Lvliang, Shanxi 033001, People’s Republic of China

## Abstract

The title compound, C_8_H_10_N_2_S, was prepared by reaction of methyl­amine solution, KOH and phenyl-iso­thio­cyanate in ethanol. It adopts a *syn*-Me and *anti*-Ph conformation relative to the C=S double bond. The dihedral angle between the N—C(=S)—N thio­urea and phenyl planes is 67.83 (6)°. In the crystal, the mol­ecules centrosymmetrical dimers by pairs of N(Ph)—H⋯S hydrogen bonds. The dimers are linked by N(Me)—H⋯S hydrogen bonds into layers parallel to (100).

## Related literature   

For applications of thio­urea derivatives, see: Madan & Taneja (1991 ▶); Xu *et al.* (2004 ▶); Borisova *et al.* (2007 ▶). For the crystal structures of related compounds, see: Ji *et al.* (2002 ▶); Wenzel *et al.* (2011 ▶).
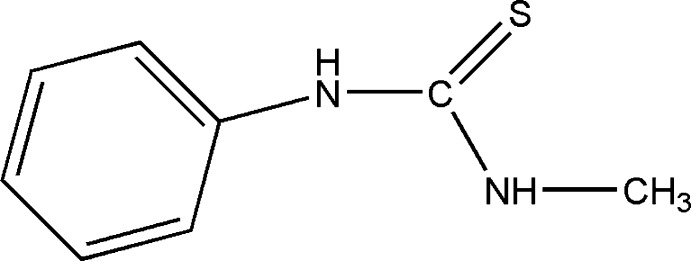


## Experimental   

### 

#### Crystal data  

C_8_H_10_N_2_S
*M* *_r_* = 166.24
Monoclinic, 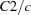

*a* = 17.348 (3) Å
*b* = 8.6023 (13) Å
*c* = 12.1672 (18) Å
β = 99.637 (3)°
*V* = 1790.1 (5) Å^3^
*Z* = 8
Mo *K*α radiation
μ = 0.30 mm^−1^
*T* = 296 K
0.25 × 0.23 × 0.20 mm

#### Data collection  

Bruker SMART CCD area-detector diffractometer
5444 measured reflections
2026 independent reflections
1424 reflections with *I* > 2σ(*I*)
*R*_int_ = 0.033

#### Refinement  

*R*[*F* ^2^ > 2σ(*F* ^2^)] = 0.041
*wR*(*F* ^2^) = 0.114
*S* = 1.03
2026 reflections
109 parameters
H atoms treated by a mixture of independent and constrained refinement
Δρ_max_ = 0.24 e Å^−3^
Δρ_min_ = −0.24 e Å^−3^

### 

Data collection: *SMART* (Bruker 1997 ▶); cell refinement: *SAINT* (Bruker 1997 ▶); data reduction: *SAINT*; program(s) used to solve structure: *SHELXS2013* (Sheldrick, 2008 ▶); program(s) used to refine structure: *SHELXL2013* (Sheldrick, 2008 ▶); molecular graphics: *SHELXTL* (Sheldrick, 2008 ▶); software used to prepare material for publication: *SHELXTL*.

## Supplementary Material

Crystal structure: contains datablock(s) I. DOI: 10.1107/S1600536814007442/kq2012sup1.cif

Structure factors: contains datablock(s) I. DOI: 10.1107/S1600536814007442/kq2012Isup2.hkl

Supporting information file. DOI: 10.1107/S1600536814007442/kq2012Isup3.cml

CCDC reference: 995308

Additional supporting information:  crystallographic information; 3D view; checkCIF report

## Figures and Tables

**Table 1:** Hydrogen-bond geometry (Å, °)

*D*—H⋯*A*	*D*—H	H⋯*A*	*D*⋯*A*	*D*—H⋯*A*
N1—H1⋯S1^i^	0.81 (2)	2.55 (2)	3.351 (2)	169 (2)
N2—H2⋯S1^ii^	0.77 (2)	2.78 (2)	3.4229 (19)	142 (2)

Symmetry codes: (i) 
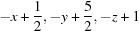
; (ii) 
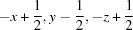
.

**Fig. 1.**
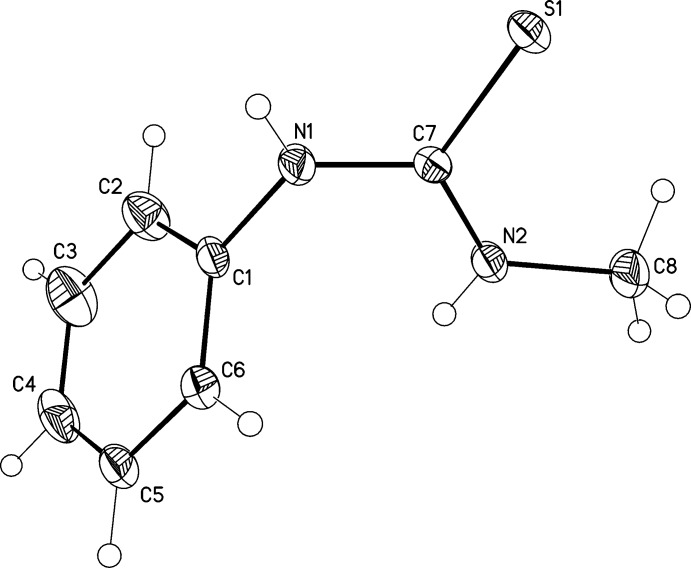
Molecular structure of I. Displacement ellipsoids are presented at the 40% probability level. H atoms are depicted as small spheres of arbitrary radius.

**Fig. 2.**
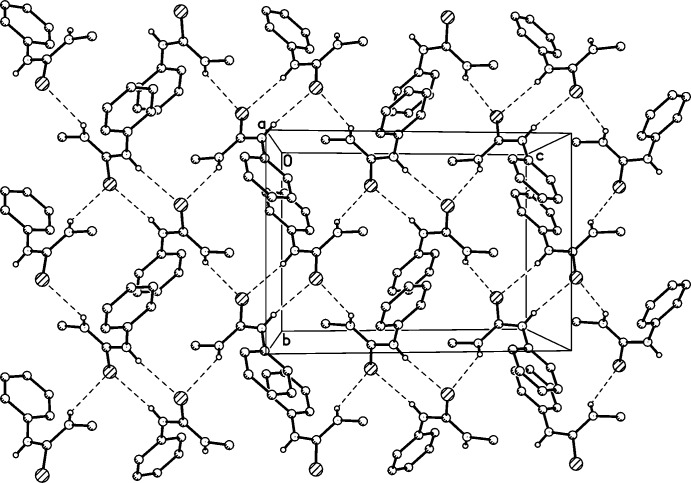
A portion of the crystal structure of I demonstrating the H–bonded dimers and layers parallel to (100). The hydrogen atoms participating in the formation of hydrogen bonds are shown only. The intermolecular N—H···S hydrogen bonds are depicted by dashed lines.

## supplementary crystallographic information

### 1. Comment

Thioureas have been studied for many years because of their broad antibiosis and sterilibzation properties. Recent years study shows that thioureas not only can be used to kill insects and adjust plant growth but also have anti-viral activities (Madan & Taneja, 1991; Borisova *et al.*, 2007). From our early quantum study on these compounds we find that they have several active centers and cart form polyligand complexes with metals easily (Xu *et al.*, 2004). These complexes are widely used as anti-medicines. Therefore study on thioureas has important impact on the future. In order to search for new compounds with higher bioactivity, the title compound was synthesized.

In the title compound, C_8_H_10_N_2_S (I), the bond lengths and angles are in a good agreement with those found in the related compounds (Ji *et al.*, 2002; Wenzel *et al.* 2011). Compound I adopts a *cis*-Me and *trans*-Ph conformation relative to the C═S double bond (Figure 1). The dihedral angle between the N1—C7(═S1)—N1 thiourea and phenyl planes is 67.83 (6)°.

In the crystal, the molecules of I form centrosymmetrical dimers by the two intermolecular N1—H1···S1^i^ hydrogen bonds (Table 1, Figure 2). The dimers are further bound to each other by the intermolecular N2—H2···S1^ii^ hydrogen bonds (Table 1) into layers parallel to (100) (Figure 2). Symmetry codes: (i) –*x* + 1/2, –*y* + 5/2, –*z* + 1; (ii) –*x* + 1/2, *y*–1/2, –*z* + 1/2.

### 2. Experimental

The title compound was prepared by reaction of methylamine solution (40%, 0.05 mol, 5.5 ml), KOH (0.15 mol, 8.4 g) and phenyl-isothiocyanate(0.05 mol, 4.65 g) in the ethanol solution (40 ml) at room temperature. Single-crystals of the title compound suitable for X-ray measurements was obtained by recrystallization from ethanol/acetone (*v*/*v*=1:1) at room temperature.

### 3. Refinement

The hydrogen atoms of the amino groups were localized in the difference Fourier map and refined isotropically. The other hydrogen atoms were placed in the calculated positions with C—H = 0.93 Å (aryl–H) and 0.96 Å (methyl–H) and refined in the riding model with fixed isotropic displacement parameters: *U*_iso_(H) = 1.5*U*_eq_(C) for the CH_3_ group and 1.2*U*_eq_(C) for the other CH groups.

### Crystal data

**Table:**

C_8_H_10_N_2_S	*F*(000) = 704
*M**_r_* = 166.24	*D*_x_ = 1.234 Mg m^−^^3^
Monoclinic, *C*2/*c*	Mo *K*α radiation, λ = 0.71073 Å
*a* = 17.348 (3) Å	Cell parameters from 1286 reflections
*b* = 8.6023 (13) Å	θ = 2.4–24.8°
*c* = 12.1672 (18) Å	µ = 0.30 mm^−^^1^
β = 99.637 (3)°	*T* = 296 K
*V* = 1790.1 (5) Å^3^	Bar, colorless
*Z* = 8	0.25 × 0.23 × 0.20 mm

### Data collection

**Table:**

Bruker SMART CCD area-detector diffractometer	*R*_int_ = 0.033
Radiation source: sealed tube	θ_max_ = 27.5°, θ_min_ = 2.4°
phi and ω scans	*h* = −22→21
5444 measured reflections	*k* = −8→11
2026 independent reflections	*l* = −15→15
1424 reflections with *I* > 2σ(*I*)	

### Refinement

**Table:**

Refinement on *F*^2^	Primary atom site location: structure-invariant direct methods
Least-squares matrix: full	Secondary atom site location: difference Fourier map
*R*[*F*^2^ > 2σ(*F*^2^)] = 0.041	Hydrogen site location: difference Fourier map
*wR*(*F*^2^) = 0.114	H atoms treated by a mixture of independent and constrained refinement
*S* = 1.03	*w* = 1/[σ^2^(*F*_o_^2^) + (0.0587*P*)^2^ + 0.1272*P*] where *P* = (*F*_o_^2^ + 2*F*_c_^2^)/3
2026 reflections	(Δ/σ)_max_ < 0.001
109 parameters	Δρ_max_ = 0.24 e Å^−^^3^
0 restraints	Δρ_min_ = −0.24 e Å^−^^3^

### Special details

**Table:**

Geometry. All e.s.d.'s (except the e.s.d. in the dihedral angle between two l.s. planes) are estimated using the full covariance matrix. The cell e.s.d.'s are taken into account individually in the estimation of e.s.d.'s in distances, angles and torsion angles; correlations between e.s.d.'s in cell parameters are only used when they are defined by crystal symmetry. An approximate (isotropic) treatment of cell e.s.d.'s is used for estimating e.s.d.'s involving l.s. planes.

### Fractional atomic coordinates and isotropic or equivalent isotropic displacement parameters (Å^2^)

**Table:**

	*x*	*y*	*z*	*U*_iso_*/*U*_eq_	
S1	0.16423 (3)	1.19173 (6)	0.35515 (4)	0.04468 (19)	
N1	0.29456 (9)	1.0463 (2)	0.43958 (15)	0.0467 (5)	
N2	0.23002 (10)	0.9493 (2)	0.27416 (15)	0.0485 (5)	
C1	0.35645 (10)	0.9351 (2)	0.45446 (17)	0.0401 (5)	
C2	0.36233 (13)	0.8311 (3)	0.5403 (2)	0.0648 (7)	
H2A	0.3251	0.8303	0.5871	0.078*	
C3	0.42442 (15)	0.7264 (3)	0.5572 (3)	0.0814 (9)	
H3	0.4287	0.6555	0.6156	0.098*	
C4	0.47899 (13)	0.7275 (3)	0.4884 (3)	0.0683 (7)	
H4	0.5202	0.6569	0.4997	0.082*	
C5	0.47334 (12)	0.8309 (3)	0.4037 (2)	0.0646 (7)	
H5	0.5109	0.8317	0.3573	0.077*	
C6	0.41193 (11)	0.9357 (3)	0.38576 (18)	0.0510 (5)	
H6	0.4082	1.0064	0.3273	0.061*	
C7	0.23382 (9)	1.0531 (2)	0.35543 (15)	0.0353 (4)	
C8	0.16755 (12)	0.9440 (3)	0.1781 (2)	0.0685 (7)	
H8A	0.1740	1.0278	0.1284	0.103*	
H8B	0.1180	0.9540	0.2026	0.103*	
H8C	0.1695	0.8467	0.1401	0.103*	
H1	0.2993 (12)	1.117 (3)	0.4843 (19)	0.054 (7)*	
H2	0.2646 (12)	0.893 (3)	0.2769 (18)	0.052 (7)*	

### Atomic displacement parameters (Å^2^)

**Table:**

	*U*^11^	*U*^22^	*U*^33^	*U*^12^	*U*^13^	*U*^23^
S1	0.0346 (3)	0.0467 (3)	0.0524 (3)	0.0114 (2)	0.0062 (2)	0.0000 (2)
N1	0.0401 (9)	0.0497 (11)	0.0462 (11)	0.0157 (8)	−0.0043 (8)	−0.0147 (9)
N2	0.0365 (9)	0.0573 (12)	0.0482 (11)	0.0134 (8)	−0.0034 (8)	−0.0132 (9)
C1	0.0293 (8)	0.0418 (11)	0.0457 (11)	0.0061 (8)	−0.0044 (8)	−0.0085 (9)
C2	0.0512 (13)	0.0680 (17)	0.0758 (17)	0.0096 (11)	0.0123 (12)	0.0206 (13)
C3	0.0690 (17)	0.0612 (18)	0.109 (2)	0.0133 (14)	0.0015 (16)	0.0307 (16)
C4	0.0427 (12)	0.0587 (16)	0.097 (2)	0.0177 (11)	−0.0081 (13)	−0.0096 (15)
C5	0.0364 (10)	0.090 (2)	0.0637 (16)	0.0163 (11)	−0.0007 (10)	−0.0205 (14)
C6	0.0397 (10)	0.0647 (15)	0.0465 (12)	0.0101 (10)	0.0009 (9)	−0.0033 (11)
C7	0.0298 (9)	0.0394 (11)	0.0373 (10)	0.0024 (7)	0.0075 (8)	0.0004 (9)
C8	0.0504 (12)	0.093 (2)	0.0555 (14)	0.0138 (12)	−0.0108 (11)	−0.0244 (14)

### Geometric parameters (Å, º)

**Table:**

S1—C7	1.6964 (17)	C3—C4	1.365 (4)
N1—C7	1.342 (2)	C3—H3	0.9300
N1—C1	1.427 (2)	C4—C5	1.353 (4)
N1—H1	0.81 (2)	C4—H4	0.9300
N2—C7	1.326 (2)	C5—C6	1.384 (3)
N2—C8	1.455 (3)	C5—H5	0.9300
N2—H2	0.77 (2)	C6—H6	0.9300
C1—C2	1.366 (3)	C8—H8A	0.9600
C1—C6	1.376 (3)	C8—H8B	0.9600
C2—C3	1.393 (3)	C8—H8C	0.9600
C2—H2A	0.9300		
			
C7—N1—C1	127.17 (17)	C3—C4—H4	119.9
C7—N1—H1	117.4 (16)	C4—C5—C6	120.2 (2)
C1—N1—H1	115.2 (16)	C4—C5—H5	119.9
C7—N2—C8	123.87 (18)	C6—C5—H5	119.9
C7—N2—H2	117.0 (17)	C1—C6—C5	120.0 (2)
C8—N2—H2	119.0 (17)	C1—C6—H6	120.0
C2—C1—C6	119.74 (18)	C5—C6—H6	120.0
C2—C1—N1	119.64 (18)	N2—C7—N1	118.32 (17)
C6—C1—N1	120.57 (18)	N2—C7—S1	121.70 (15)
C1—C2—C3	119.6 (2)	N1—C7—S1	119.98 (14)
C1—C2—H2A	120.2	N2—C8—H8A	109.5
C3—C2—H2A	120.2	N2—C8—H8B	109.5
C4—C3—C2	120.2 (3)	H8A—C8—H8B	109.5
C4—C3—H3	119.9	N2—C8—H8C	109.5
C2—C3—H3	119.9	H8A—C8—H8C	109.5
C5—C4—C3	120.2 (2)	H8B—C8—H8C	109.5
C5—C4—H4	119.9		
			
C7—N1—C1—C2	−112.2 (2)	C2—C1—C6—C5	0.1 (3)
C7—N1—C1—C6	70.3 (3)	N1—C1—C6—C5	177.57 (19)
C6—C1—C2—C3	−0.1 (3)	C4—C5—C6—C1	0.2 (3)
N1—C1—C2—C3	−177.7 (2)	C8—N2—C7—N1	−179.8 (2)
C1—C2—C3—C4	−0.1 (4)	C8—N2—C7—S1	0.4 (3)
C2—C3—C4—C5	0.4 (4)	C1—N1—C7—N2	−1.9 (3)
C3—C4—C5—C6	−0.4 (4)	C1—N1—C7—S1	177.91 (16)

### Hydrogen-bond geometry (Å, º)

**Table:**

*D*—H···*A*	*D*—H	H···*A*	*D*···*A*	*D*—H···*A*
N1—H1···S1^i^	0.81 (2)	2.55 (2)	3.351 (2)	169 (2)
N2—H2···S1^ii^	0.77 (2)	2.78 (2)	3.4229 (19)	142 (2)

Symmetry codes: (i) −*x*+1/2, −*y*+5/2, −*z*+1; (ii) −*x*+1/2, *y*−1/2, −*z*+1/2.
